# Improving the outcome of salvage treatment for non-seminomatous germ cell tumours (NSGCT)

**DOI:** 10.1054/bjoc.2000.1290

**Published:** 2000-07-24

**Authors:** J S de Bono, J Paul, A Simpson, A Anthoney, D Kirk, M Underwood, J Graham, S B Kaye

**Affiliations:** Departments of Medical Oncology, University of Glasgow; Urology, Western Infirmary, Dumbarton Road, Glasgow, G11 6NT, UK

**Keywords:** teratoma, relapse, salvage, prognosis

## Abstract

Between 1991–96, 41 patients were treated in this unit for relapsed non-seminomatous germ cell tumours (NSGCT). Twenty-eight patients had raised markers at relapse: 17 required salvage chemotherapy and post-chemotherapy surgery, 11 only chemotherapy. In addition 9 patients received high dose chemotherapy. Overall 16/28 patients (57%) requiring chemotherapy remain alive, 14 (50%) disease free. Of the 17 patients treated with chemotherapy and surgery: 12 remain alive, 10 (59%) with no evaluable disease. Only 4/11 (36%) patients treated with chemotherapy alone remain alive, all in complete remission (CR). For relapse with raised markers, univariate analysis suggests that less than CR to induction therapy, resulting in the presence of residual disease is the most important predictor of poor outcome (*P*< 0.001). All of the 13 patients relapsing with normal markers remain alive, having been primarily treated surgically. Overall these results indicate an improving outlook for relapsed NSGCT. © 2000 Cancer Research Campaign


*British Journal of Cancer* (2000) **83**(4), 426–430
© 2000 Cancer Research Campaign
doi: 10.1054/ bjoc.2000.1290, available online at http://www.idealibrary.com on IDEAL®

### Improving the outcome of salvage treatment for non-seminomatous germ cell tumours (NSGCT)

**JS de Bono[1], J Paul[1], A Simpson[1], A Anthoney[1], D Kirk[2], M Underwood[2], J Graham[2] and SB Kaye[1]**

University of Glasgow, [1]Departments of Medical Oncology and [2]Urology, Western Infirmary, Dumbarton Road, Glasgow G11 6NT, UK

**Summary** Between 1991–96, 41 patients were treated in this unit for relapsed non-seminomatous germ cell tumours (NSGCT). Twenty-eight patients had raised markers at relapse: 17 required salvage chemotherapy and post-chemotherapy surgery, 11 only chemotherapy. In addition 9 patients received high dose chemotherapy. Overall 16/28 patients (57%) requiring chemotherapy remain alive, 14 (50%) disease free. Of the 17 patients treated with chemotherapy and surgery: 12 remain alive, 10 (59%) with no evaluable disease. Only 4/11 (36%) patients treated with chemotherapy alone remain alive, all in complete remission (CR). For relapse with raised markers, univariate analysis suggests that less than CR to induction therapy, resulting in the presence of residual disease is the most important predictor of poor outcome ($P < 0.001$). All of the 13 patients relapsing with normal markers remain alive, having been primarily treated surgically. Overall these results indicate an improving outlook for relapsed NSGCT. © 2000 Cancer Research Campaign

**Keywords**: teratoma; relapse; salvage; prognosis

Approximately 20–30% of patients with metastatic germ cell tumours will not achieve durable remissions after first line treatment (International Germ Cell Cancer Collaborative Group, 1997). Most suffer relapses or disease progression after a favourable initial response. A variety of salvage chemotherapy regimens have been used, usually involving a combination of cisplatin, ifosfamide and perhaps etoposide or vinblastine (Loehrer et al, 1998). More recently paclitaxel (Motzer et al, 1994) and gemcitabine (Bokemeyer et al, 1999) have been shown to have significant activity. Paclitaxel is therefore currently being investigated in combination with ifosfamide and cisplatin as first line salvage therapy (Motzer et al, 1998). High dose chemotherapy and stem-cell transplantation has also been successfully utilized for these patients (Bhatia et al, 1998).

The essential role of surgery in the treatment of relapsed teratoma is well described (Jansen et al, 1991). Surgery is usually the treatment of choice in relapse with normal markers. Salvage surgery is also vital for post-salvage chemotherapy masses, and can be curative when markers are rising despite salvage chemotherapy (Murphy et al, 1993). If relapse occurs late (several years following initial therapy), in the absence of extensive recurrence or raised markers, salvage surgery may be the primary mode of treatment (Baniel et al, 1995).

Overall the results obtained with salvage therapy remain poor. Reported series indicate that less than 50% of patients will achieve a favourable response to salvage chemotherapy, with 20–25% remaining disease free (Loehrer et al, 1998). While the prognosis of these patients remains unsatisfactory, however, our impression is that improvements are being made. Our aim has been to report our most recent experience, and identify prognostic factors for outcome of relapse.

*Received 13 August 1999*
*Revised 28 February 2000*
*Accepted 13 April 2000*

*Correspondence to:* JS de Bono

### PATIENTS AND METHODS

This retrospective study comprised all patients treated for NSGCT between 1991 and 1996 at the Beatson Oncology Centre, Glasgow. Patient identification was based on West of Scotland cancer registration. Data were abstracted directly from the patient records. All patients had received platinum-based primary combination chemotherapy. The majority of patients had received BEP (bleomycin, etoposide and cisplatin), or for poorer prognosis patients BOP-VIP (Kaye et al, 1998) or BOP-BEP (Anthoney et al, 1999) in clinical trials. Eighteen patients had received carboplatin-based chemotherapy within a Medical Research Council (MRC) randomized trial for good prognosis patients (Horwich et al, 1997). Patients with residual post-chemotherapy masses had undergone surgery to remove them by a specific specialist surgical team with an interest in this tumour type. If residual malignant disease was identified in the post-chemotherapy resection specimen additional chemotherapy was usually given in the form of either further cisplatin-based combination chemotherapy or oral etoposide.

Patients were defined as having achieved CR if, after chemotherapy, tumour markers and CT scan were normal. Patients who were marker negative after chemotherapy, and who subsequently underwent complete surgical resection of carcinoma or teratoma were classified as no evidence of disease $(NED)_{carcinoma}$ or $NED_{teratoma}$ respectively. Patients with incompletely resected residual masses were described as having residual carcinoma or teratoma.

Recurrence was defined on the basis of rising tumour marker (HCG and/or αFP) and/or the development of new or enlarging metastases on plain radiographs or computerised tomography (CT). The salvage treatment utilised depended on the presentation at relapse and previous treatment. Patients with radiological evidence of a new lesion but normal markers were managed by initial surgical resection and did not receive chemotherapy unless malignant teratoma was seen in the resected specimens. Patients with rising tumour markers were treated with chemotherapy initially. The commonest chemotherapy regimen used was VIP: etoposide, ifosfamide and cisplatin. A number of other regimens were used including VelP; vinblastine, ifosfamide and cisplatin: weekly cisplatin and etoposide; and POMB-ACE: cisplatin, vincristine, methotrexate, bleomycin, actinomycin D, cyclophosphamide and etoposide (Bower et al, 1997). The majority of these patients received between 3 and 6 cycles of chemotherapy (median 4 cycles). Salvage surgery to resect any residual disease was performed, if necessary, after the completion of chemotherapy. Radiotherapy was utilized primarily following resection of cerebral metastases, although it was also successfully used in one patient who relapsed with extensive penile, scrotal and inguinal disease. A further 3 patients with incomplete resection of para-aortic carcinomatous disease were treated with radiotherapy to the tumour bed. This was not standard practice and was only utilized in the absence of other treatment options.

Patients relapsing with rising markers, with evidence of further response to conventional dose regimes, were considered for entry into a non-randomised trial of high dose chemotherapy with peripheral blood stem cell transplantation. High dose chemotherapy comprised carboplatin (total dose was AUC 20 given over 4 days: d1–4), cyclophosphamide (total dose: 120 mg/kg divided over d1 and d3), and etoposide (total dose 1.2 g/m$^2$ given over 4 days: d1–4).

After salvage treatment patients were followed up at 2 to 3 monthly intervals in the first year, then 4 to 6 monthly in subsequent years, with clinical, radiological and biochemical examination (αFP, HCG, LDH). Survival times were measured from the date of diagnosis of progression to the date of death or date last seen.

Survival times were calculated from the date of diagnosis of relapse to date of death or date last seen. The Mantel-Haenszel logrank test was used to examine the prognostic value of the variables in Table 5. Multivariate analyses were not performed because of the small number of events involved. Kaplan-Meier estimates were used for calculating the percent alive at 2 years in the same table and for drawing the survival curves.

### RESULTS

Forty-one patients were treated for relapsed teratoma between 1991 and 1996. Twenty-eight of 41 patients had raised markers at relapse. The features of these 28 patients at primary diagnosis and at relapse are presented in Tables 1 and 2. Their median age at relapse was 29 years, with a median time to relapse of 13 months (range: 7 to 121 months). Twelve of these 28 patients have died following treatment for subsequent relapse (Figure 1), 14 (50%) remain alive with NED. For the 16 living patients the median follow-up is 72 months (range 26 to 121 months). Table 3 describes the treatment of relapsing disease: all 28 patients were treated with chemotherapy, with 17 patients having post-chemotherapy resection of operable residual disease (Table 3). Of these 17 patients treated with chemotherapy and surgery, 10 remain alive with NED (59%), 2 remain alive with residual disease, and 5 have died. The other 11 patients were treated with chemotherapy alone: only 4 (36%) of these patients remain alive in CR, the rest having died of progressing disease.

An additional 13 patients developed recurrent disease with normal markers. They were primarily treated surgically (Table 4). These 13 patients all remain alive with NED, and are not included in the prognostic factor univariate analyses.

### Prognostic factors associated with relapse

A recent study by Fossa et al (1999) identified 3 main factors predicting a poor prognosis at relapse. These included progression-free interval (<2 years vs >2 years), response to induction

**Table 1** Characteristics, at initial diagnosis, of patients relapsing with raised markers ($n$ = 28)

| Characteristic | Group | Number of patients (% of total) |
|---|---|---|
| Primary site | Testicular | 25 (89) |
| | Extragonadal | 3 (11) |
| Prognostic group at initial diagnosis (IGCCCG) | Good | 15 (50) |
| | Intermediate | 3 (11) |
| | Poor | 10 (39) |
| Primary chemotherapy | BEP | 14 (50) |
| | CEB | 4 (14) |
| | EP | 2 (7) |
| | BOP-BEP | 5 (18) |
| | BOP-VIP | 2 (7) |
| | PVB | 1 (4) |
| Response to primary chemotherapy. | Complete remission | 12 (43) |
| | Residual disease | 16 (57) |
| Surgery post-primary chemotherapy (Patients can have surgery to more than one site) | Operable residual disease | 15 |
| | Para-aortic nodes | 11 |
| | Pulmonary metastases | 6 |
| | Supraclavicular nodes | 3 |
| | Brain metastases | 1 |
| | Inoperable residual disease (liver metastases: malignant on biopsy) | 1 |
| Resection margins at above surgery | Complete resection | 10 |
| | Incomplete resection | 5 |
| Pathology of resected mass at above surgery | Necrosis | 5 |
| | Mature teratoma | 3 |
| | Viable malignant disease | 7 |

**Table 2** Characteristics, at relapse, of patients relapsing with raised markers ($n$ = 28)

| | | |
|---|---|---|
| Patients | | 28 |
| Age | Median | 29 years |
| | Range | 19–57 years |
| Remission status obtained after initial therapy | Complete remission | 12 |
| | Partial remission post-chemotherapy and: | |
| | a) necrosis at surgery | 5 |
| | b) no evaluable disease $(NED)_{teratoma}$ | 3 |
| | c) $NED_{carcinoma}$ | 1 |
| | d) residual malignant disease | 7 |
| Disease free interval | <3 months | 6 |
| | ≥3 months but <2 years | 17 |
| | ≥2 years but <5 years | 3 |
| | ≥5 years | 2 |
| Markers at Relapse | Low[a] | 14 |
| | High | 14 |

[a]αFP< 100ku/L; HCG< 100 iu/L; LDH < 1.5 × normal (17).

**Table 3** Treatment at relapse of patients relapsing with raised markers ($n$ = 28)

| | | |
|---|---|---|
| Treatment of relapse | Chemotherapy alone | 11 (39%) |
| | Chemotherapy and surgery | 17 (61%) |
| Relapse chemotherapy | VP-16, Ifosfamide, Cisplatin (VIP) | 16 |
| | Taxol, ifosfamide, cisplatin (TIP) | 2 |
| | Vinblastine, ifosfamide, cisplatin (VnIP) | 1 |
| | POMB-ACE | 2 |
| | Other | 7 |
| High dose chemotherapy and PBSCT | Carboplatin, VP-16 and cyclophosphamide | 9 |
| Sites of surgery following salvage chemotherapy | Para-aortic nodes | 8 |
| | Pulmonary | 7 |
| | Brain | 3 |
| | Retrocrural | 0 |
| | Pelvic mass | 2 |
| | Mediastinum | 1 |
| | Scrotal mass | 1 |
| | Supraclavicular mass | 1 |
| Pathology of surgery | Surgical resection margins | Pathology |
| | Complete 9 | Necrosis 2 |
| | Incomplete 5 | Mature teratoma 5 |
| | Equivocal 3 | Malignant 10 |
| Post-operative chemotherapy | 3 cycles of oral VP-16-100 mg/day, 14 days q21 if resection pathology revealed carcinoma. | 5 |
| Post-operative radiotherapy | Para-aortic nodes[a] | 3 |
| | Brain | 3 |
| | Inguinal and scrotal[a] | 1 |
| Status | Alive in CR | 14 (54%) |
| | Alive with residual disease. | 2 (3%) |
| | Dead | 12 (43%) |

[a]Radiotherapy was only used when this was the only treatment option available to palliate disease.

therapy (CR vs not in CR) and level of serum HCG and αFP at relapse (<100 or >100). We performed a univariate analysis of these 3 potential prognostic factors, the results of which are shown in Table 5. This study confirms that an incomplete response to primary treatment, resulting in residual unresected disease, is the most important prognostic parameter. Progression free interval and level of tumour markers at relapse were not found to be independent prognostic factors in our study. When these three prognostic factors are used together, however, patients with 0–1 of these risk factors were found to have a significantly better outcome than those with 2–3 risk factors ($P$ = 0.0027).

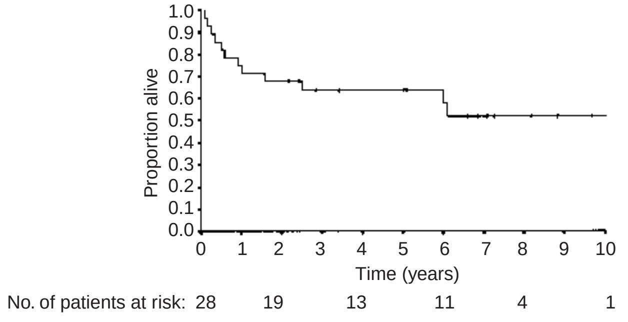

**Fig.1** Survival curve of patients relapsing with raised markers ($n$=28)

 *© 2000 Cancer Research Campaign*

**Table 4** Characteristics of patients relapsing with normal markers (*n* = 13)

| Relapse surgery | Primary salvage surgery alone |
|---|---|
| Total number of patients | 13 |
| Sites of relapse surgery | (3 had surgery at >1 site) |
| Para-aortic nodes | 6 |
| Pulmonary | 4 |
| Brain | 2 |
| Retrocrural | 1 |
| Pelvic mass | 1 |
| Mediastinum | 1 |
| Scrotal mass | 0 |
| Supraclavicular mass | 0 |
| Resection margins | |
| Complete | 11 |
| Incomplete | 0 |
| Equivocal | 2 (brain metastases) |
| Pathology | |
| Necrosis | 0 |
| Mature teratoma | 10 |
| Malignant | 3[a] |
| Therapy post surgery[a] | |
| Radiotherapy | 2 |
| Chemotherapy | 1 (Oral VP-16: 12 weeks) |

[a]Two patients had cerebral metastasis and received post-resection radiotherapy; one had a small focus of malignant disease, in largely mature teratoma, which was completely resected: he received post-resection oral VP-16 for 12 weeks.

**Table 5** Univariate analyses of prognostic factors identified by Fossa et al (1999)

| Variable | Categories | No. of patients (% alive at 2 years) | Logrank P-value |
|---|---|---|---|
| Achieved CR–radiological or pathological (necrosis at surgery to initial chemotherapy | Yes | 17 (94) | <.001 |
| | No | 11 (13) | |
| High markers at relapse (HCG > 100 iu/L; αFP>100 ku/L; LDH<1.5 × normal | Yes | 14 (50) | 0.19 |
| | No | 14 (86) | |
| Progression free interval | <2 years | 23 (70) | 0.84 |
| | ≥2 years | 5 (60) | |
| No. of above risk factors | 0/1 | 12 (100) | 0.0027 |
| | 2/3 | 16 (44) | |

### DISCUSSION

A widely accepted prognostic factor based staging system to guide the treatment of NSGCT patients progressing after induction cisplatin-based chemotherapy has not yet been defined. A recent study analysing 164 relapsing patients described an overall survival rate of 30% (Fossa et al, 1999). This study identified a number of poor prognostic factors and indicated that treatment in a large specialist centre could improve outcome.

Indeed our findings do suggest that the outcome of relapsed NSGCT is improving, and confirm the prognostic significance of the factors identified by Fossa et al (1999). There are a number of potential reasons for this improvement in outcome. Firstly, 57% (94 of 164 patients) of the relapse patients described by Fossa et al were treated for relapse prior to 1986. Secondly, management of NSGCT has been shown to have a better outcome in a larger specialist treatment centre (Harding et al, 1993; Collette et al, 1999). Thirdly, since surgery is a key component of salvage therapy, access to a specialist surgical team with an interest in NSGCT is crucial. These results therefore have implications for future decisions regarding patterns of health care. We propose that the management of relapsed NSGCT should be carried out with curative intent, in a specialist unit, with access to a dedicated specialist surgical team.

### REFERENCES

Anthoney DA, Roberts J, Graham J, McKean MJ, Paul J and Kaye SB (1999) Alternating Bleomycin, Vincristine, Cisplatin (BOP)/Bleomycin, Etoposide, Cisplatin (BEP) chemotherapy as a dose intense regimen for intermediate and poor prognosis malignant germ cell tumours (MGCT). *Proc Am Soc Clin Oncol*

Baniel J, Foster RS, Gonin R, Messemer JE, Donohue JP and Einhom LH (1995) Late relapse of testicular cancer. *J Clin Oncol* **13(5):** 1170–1176

Bhatia S, Cornetta K, Broun R, Nichols C, Abonour R and Einhom LH (1998) High dose chemotherapy with peripheral stem cell or autologous bone marrow transplant as initial salvage chemotherapy for testicular cancer. *Proc Am Soc Clin Oncol*

© 2000 Cancer Research Campaign 

Bokemeyer C, Gerl A, Schoffski P, Harstrick A, Niederle N, Beyer J, Casper J, Schmoll HJ and Kanz L (1999) Gemcitabine in patients with relapsed or cisplatin-refractory testicular cancer. *J Clin Oncol* **17(2):** 512–516

Bower M, Newlands ES, Holden L, Rustin GJ and Begent RH (1997) Treatment of men with metastatic non-seminomatous germ cell tumours with cyclical POMB/ACE chemotherapy. *Ann Oncol* **8(5):** 477–483

Collette L, Sylvester RJ, Stenning SP, Fossa SD, Mead GM, de Wit R, de Mulder PH, Neymark N, Lallemand E and Kaye SB. (1999) Impact of the treating institution on survival of patients with 'poor-prognosis' metastatic non-seminoma. European Organisation for Research and Treatment of Cancer Genito-Urinary Tract Cancer Collaborative Group and the Medical Research Council Testicular Cancer Working Party. *J Natl Cancer Inst* **91(10):** 839–846

Fossa SD, Stenning S, Gerl A, Horwich A, Clark P, Wilkinson P, Jones WG, Williams M, Oliver T, Newlands E, Mead G, Cullen M, Kaye SB, Rustin G and Cook P (1999) Prognostic factors in patients with malignant non-seminomatous germ cell tumours relapsing after cisplatin based chemotherapy. *Br J Cancer* **80(9):** 1392–1399

Harding MJ, Paul J, Gillis CR and Kaye SB (1993) Management of malignant teratoma: does referral to a specialist unit matter? *Lancet* **341(8851):** 999–1002

Horwich A, Sleijfer DT, Fossa SD, Kaye SB, Oliver RT, Cullen MH, Mead GM, de Wit R, de Mulder PH, Dearnaley DP, Cook PA, Sylvester RJ and Stenning SP (1997) Randomized trial of bleomycin, etoposide, and cisplatin compared with bleomycin, etoposide, and carboplatin in good-prognosis metastatic non-seminomatous germ cell cancer: a Multi-institutional Medical Research Council/European Organisation for Research and Treatment of Cancer Trial. *J Clin Oncol* **15(5):** 1844–1852

International Germ Cell Consensus Classification: a prognostic factor-based staging system for metastatic germ cell cancers. (1997) International Germ Cell Cancer Collaborative Group. *J Clin Oncol.* **15(2):** 594–603

Jansen RL, Sylvester R, Sleyfer DT, ten Bokkel Huinink WW, Kaye SB, Jones WG, Keizer J, van Oosterom AT, Meyer S, Vendrik CP, de Pauw M and Stoter G for the EORTC Genitourinary Tract Cancer Cooperative Group 1991 Long-term follow-up of non-seminomatous testicular cancer patients with mature teratoma or carcinoma at postchemotherapy surgery. *Eur J Cancer* **27(6):** 695–698

Kaye SB, Mead GM, Fossa S, Cullen M, deWit R, Bodrogi I, van Groeningen C, Sylvester R, Collette L, Stenning S, De Prijck L, Lallemand E, deMulder P (1998) Intensive induction-sequential chemotherapy with BOP/VIP-B compared with treatment with BEP/EP for poor-prognosis metastatic non-seminomatous germ cell tumour: a Randomized Medical Research Council/European Organisation for Research and Treatment of Cancer study. *J Clin Oncol.* **16(2):** 692–701

Loehrer PJ, Gonin R, Nichols CR, Weathers T and Einhorn LH (1998) Vinblastine plus ifosfamide plus cisplatin as initial salvage therapy in recurrent germ cell tumor. *J Clin Oncol* **16:** 2500–2504

Motzer RJ, Bajorin DF, Schwartz LH, Hutter HS, Bosl GJ, Scher HI, Lyn P and Fischer P (1994) Phase II trial of paclitaxel shows anti-tumour activity in patients with previously treated germ cell tumours. *J Clin Oncol.* **12(11):** 2277–2283

Motzer RJ, GA, Green, McCaffrey JA, Bajorin DF, Bosl GJ, Sheinfeld PC and Sogani (1998) Paclitaxel (t), Ifosfamide (i), and Cisplatin (p) as first-line salvage therapy for relapsed germ cell tumour patients with favourable prognostic features. *Proc Am Soc Clin Oncol.*

Murphy BR, Breeden ES, Donohue JP, Messemer J, Walsh W, Roth BJ and Einhorn LH (1993) Surgical salvage of chemorefractory germ cell tumors. *J Clin Oncol* **11(2):** 324–329

 *© 2000 Cancer Research Campaign*
